# CD34 expression modulates tube-forming capacity and barrier properties of peripheral blood-derived endothelial colony-forming cells (ECFCs)

**DOI:** 10.1007/s10456-016-9506-9

**Published:** 2016-04-04

**Authors:** Dimitar Tasev, Lara S. F. Konijnenberg, Joana Amado-Azevedo, Michiel H. van Wijhe, Pieter Koolwijk, Victor W. M. van Hinsbergh

**Affiliations:** Department of Physiology, Institute for Cardiovascular Research, VU University Medical Center Amsterdam, De Boelelaan 1118, 1081 HV Amsterdam, The Netherlands; A-Skin Nederland BV, De Boelelaan 1117, 1007 MB Amsterdam, The Netherlands

**Keywords:** Endothelial colony-forming cells, CD34, Angiogenesis, Barrier function

## Abstract

Endothelial colony-forming cells (ECFC) are grown from circulating CD34^+^ progenitors present in adult peripheral blood, but during in vitro expansion part of the cells lose CD34. To evaluate whether the regulation of CD34 characterizes the angiogenic phenotypical features of PB-ECFCs, we investigated the properties of CD34^+^ and CD34^−^ ECFCs with respect to their ability to form capillary-like tubes in 3D fibrin matrices, tip-cell gene expression, and barrier integrity. Selection of CD34^+^ and CD34^−^ ECFCs from subcultured ECFCs was accomplished by magnetic sorting (FACS: CD34^+^: 95 % pos; CD34^−^: 99 % neg). Both fractions proliferated at same rate, while CD34^+^ ECFCs exhibited higher tube-forming capacity and tip-cell gene expression than CD3^4−^ cells. However, during cell culture CD34^−^ cells re-expressed CD34. Cell-seeding density, cell–cell contact formation, and serum supplements modulated CD34 expression. CD34 expression in ECFCs was strongly suppressed by newborn calf serum. Stimulation with FGF-2, VEGF, or HGF prepared in medium supplemented with 3 % albumin did not change CD34 mRNA or surface expression. Silencing of CD34 with siRNA resulted in strengthening of cell–cell contacts and increased barrier function of ECFC monolayers as measured by ECIS. Furthermore, CD34 siRNA reduced tube formation by ECFC, but did not affect tip-cell gene expression. These findings demonstrate that CD34^+^ and CD34^−^ cells are different phenotypes of similar cells and that CD34 (1) can be regulated in ECFC; (2) is positively involved in capillary-like sprout formation; (3) is associated but not causally related to tip-cell gene expression; and (4) can affect endothelial barrier function.

**Electronic supplementary material:**

The online version of this article (doi:10.1007/s10456-016-9506-9) contains supplementary material, which is available to authorized users.

## Introduction

Adequate regeneration of the tissue affected by ischemic insult relies on efficient angiogenesis that can be facilitated by tissue engineering or cell-based therapeutics involving endothelial cells (ECs). Endothelial cells from different vascular beds differ in their angiogenic ability to induce neovascularization in vivo [1]. Therefore, selection of an adequate EC type is a prerequisite step for successful construction of tissue-engineered vascular constructs.

Endothelial colony-forming cells (ECFCs) are considered as the most suitable ECs type for regenerative angiogenesis [2]. These cells, also named as late outgrowth endothelial progenitor cells (EPCs) or blood-outgrowth endothelial cells (BOECs), differ from the early outgrowth CD14^+^/CD45^+^ EPC which originate from the myeloid/monocytic lineage and participate in vascular regeneration in a paracrine fashion [3]. The ECFCs, which can be obtained from cord or peripheral blood, exhibit pronounced vascularization ability in vivo by physically incorporating into newly formed blood vessels [4]. In particular, their neovascularization potency is accentuated in combination with stem/progenitors cells like mesenchymal stem cells or adipose-derived stem cells [5]. Previous studies revealed that ECFCs originate from the blood-derived mononuclear progenitor cell fraction expressing CD34 among other stem/progenitor cell surface markers such as CD31 or CD146 [6, 7] and are negative for CD14 or CD45.

CD34 is a widely used cell surface marker for detection and isolation of stem and progenitor cells with robust regenerative potential for therapeutic purposes [8]. CD34 is a 115-kDa transmembrane glycoprotein rich with O- and N-glycans. Depending on the cell type, specific modifications by sulfation or sialylation [9, 10] modulate the function of the CD34 structure in such manner that different ligands in different environments can interact with this surface protein [11]. Although the CD34 function remains elusive, its expression is modulated by growth factors TGF-β1 and TNF-α [12, 13], by oxygen concentration [14], as well as by several transcription factors [15, 16]. Beside on HSCs [17], it is expressed on small vessel endothelial cells [18], mast cells [19], epidermal stem cells [20] as well as tumor cells [21]. In endothelial cells CD34 has been used to identify sprouting endothelial tip cells during neovascularization in vitro [22] and in vivo [21–23]. Previous publications on other cells reported that CD34 modulates cyto-adhesion [24–26] and cell shape [27], or is critically involved in L-selectin-mediated transendothelial migration of T-lymphocytes in high venular endothelia [28, 29]. However, the function of CD34 remains still obscure.

When ECFCs are propagated in culture, they retain stable expression of endothelial specific markers on their surface, but CD34 declines during serial propagation [30]. Monolayers of peripheral blood-derived ECFCs (PB-ECFCs) comprise mixture of cells that do or do not express CD34 on cell surface implying that phenotypical differences might exist between CD34^+^ and CD34^−^ cells. Previous reports that ECFCs which originate from CD34^+^ colonies exhibit reduced vascularization properties than the cells isolated from CD34^−^ ECFCs colonies [31] further underpins the necessity to investigate the angiogenic features of CD34^+^ and CD34^−^ ECFC. It might be that the CD34 expression in ECFCs marks a specific state of an endothelial phenotype that might have different angiogenic properties.

Therefore, we investigated whether the regulation of CD34 characterizes the angiogenic phenotypical features of PB-ECFCs. Findings presented in this study revealed that the CD34 expression is inducible and regulated in PB-ECFCs by the state of confluency and serum factors in the growth medium. By separation of CD34^+^ and CD34^−^ cells and silencing of CD34 with siRNA, we subsequently investigated the contribution of CD34 during the formation of endothelial tubules in a 3D-fbrin matrix, the expression of tip-cell-associated genes, and the endothelial barrier function of ECFCs’ monolayers.

## Materials and methods

### Cell culture

Peripheral blood-derived ECFCs were isolated, expanded, and the endothelial-phenotype-confirmed as previously described [30]. Briefly, the mononuclear cells (MNC) obtained from adult peripheral blood were plated in EGM-2 medium (Lonza, Walkersville, MD, USA) supplemented with 10 % platelet lysate (PL-EGM) and after 10 days the first ECFC colonies emerged. The isolated cells were immunophenotypically characterized by flow cytometry (FC) and were positive for CD31, vWF, CD34, CD144, CD309, and negative for CD14, CD45, and CD133 [30]. For experimental purposes, the cells were also expanded for 1–2 passages in complete medium (CMi) composed of M199 (Lonza, Verviers, Belgium) supplemented, 10 % newborn calf serum (NBCS), 10 % human serum (HS), 20 µg/mL endothelial cell-growth factor, 2 mM l-glutamine, penicillin/streptomycin (100 U/mL/100 µg/mL), and 5 U/mL heparin.

### Magnetic separation and tube formation assay

Separation of PB-ECFCs on CD34^+^ and CD34^−^ cells was performed using CD34 MicroBead Kit (clone QBEND/10, #130-046-703, Miltenyi Biotech BV, the Netherlands) according manufacturers’ protocol.

To increase the purity of yielded CD34 positive and negative fractions, the cells were twice passed through LS columns and the efficiency of separation was evaluated by flow cytometry (FC) using PE or FITC labeled mouse anti-human CD34 antibody which recognizes different epitope than QBend-10 (clone 581, #555822, BD Biosciences, the Netherlands). Data were analyzed using the FCS Express 4 software package (DeNovo Software, Toronto, Ontario, Canada). The efficiency of separation was also evaluated by determination of the mRNA levels of CD34 in positive and negative fractions by qRT-PCR. After separation, the CD34^+^ and CD34^−^ cells were used for experimental purposes. For assessment of sprouting ability of CD34^+^ and CD34^−^ PB-ECFCs, 20,000 cells positive or negative for CD34 antigen were seeded on 3D human fibrin matrices prepared as previously described [32]. Following overnight incubation in M199 (Lonza, Verviers, Belgium) supplemented with 10 % inactivated human serum and 10 % newborn calf serum, tube formation was induced by stimulating the cells with combination of 25 ng/mL vascular endothelial factor-A (VEGF-A) + 10 ng/mL fibroblast growth factor-2 (FGF-2). All growth factors were purchased from RELIATech GmbH, Wolfenbuttel, Germany. After 48-h stimulation, the cells were fixed with 2 % paraformaldehyde/HBSS, and quantification of the length of formed tube-like structures was performed using Optimas image analysis software as previously described [32]. The tube formation ability of PB-ECFCs of three different donors was each determined in triplicate wells.

### Proliferation ability of CD34^+^ and CD34^−^ cells

For short-term proliferation assays 500 CD34^+^ or CD34^−^ cells/cm^2^ were seeded onto 12-well plate precoated with rat-tail collagen type I (BD Biosciences, Erebodegem, Belgium) in PL-EGM. Renewal of culture medium was performed every other day. After 7 days in culture, the cells were washed with PBS and fixed with pre-warmth 2 % paraformaldehyde/HBSS. DAPI Vectashield Hardset (Vector Laboratories Ltd, BrunschwigChemie, the Netherlands) was used to visualize the cell nuclei, and five pictures from each well were taken using phase contrast microscopy. The number of cells was determined using ImageJ software, and the calculations for population doubling was performed as previously described [33].

### Re-expression of CD34 during cell culture

To investigate whether CD34 is re-expressed in CD34^−^ PB-ECFCs that were obtained after the magnetic separation from the cell cultures of four different donors, the CD34^−^ cells were seeded in CMi medium (CD34^−^ CMi cells) or in PL-EGM medium (CD34^−^ PL cells) and left to reach confluence (Online resource, Supp. Figure 1).

Part of CD34^−^ PB-ECFCs was also incubated in M199 supplemented with 3 % pyrogen-free human serum albumin (3 % HSA, Sanquin, Amsterdam, the Netherlands) for 24 h. The CD34 expression was evaluated by FC. After 3–4 days in cell culture CD34^−^ CMi cells and CD34^−^ PL cells were harvested for experimental purposes. The CD34 expression in CD34^−^ CMi cells and CD34^−^ PL cells was evaluated by qRT-PCR and FC. To investigate the expression of tip-cell-associated genes in CD34^−^ PL cells that re-express CD34, the CD34^−^ PL ECFCs were separated on CD34^+^ and CD34^−^ cells by magnetic separation as previously described and qRT-PCR was performed.

### Effect of serum supplements and growth factors on CD34 expression in PB-ECFCs

For assessment of the effect of NBCS on CD34 expression in PB-ECFCs over period of 24 h, the cells were incubated with 10 % NBCS, 10 % PL, 10 % NBCS + 10 % PL, and 3 % HSA all prepared in M199. The cells were harvested after 9-, 12-, and 24-h incubation period, and the CD34 mRNA levels were evaluated by qRT-PCR.

To investigate the expression of CD34 in cell cultures of PB-ECFCs in CMi and PL-EGM, the ECFCs obtained from different donors were expanded for two passages in CMi or PL-EGM medium, and the CD34 surface expression was evaluated by FC.

The effect of pro-angiogenic growth factors and 10 % PL on CD34 expression in PB-ECFCs was investigated after 24-h incubation of the cells with 10 ng/mL fibroblast growth factor-2 (FGF-2, 25 ng/mL), vascular endothelial factor-A (VEGF-A, 10 ng/mL), hepatocyte growth factor(HGF, 10 ng/mL) and 10 % PL all prepared in M199 supplemented with 3 % HSA. In parallel the cells were also incubated with 10 ng/mL FGF-2, 25 ng/mL VEGF-A, 10 ng/mL HGF, and 10 ng/mL TNF-α all prepared in EBM-2 medium supplemented with 5 % PL for 24 h. Cells incubated only in M199 + 3 % HSA or EBM-2 + 5 % PL served as control. The mRNA and surface expression of CD34 was measured by qRT-PCR and FC.

### Effect of cell-seeding density and proliferation on CD34 expression in PB-ECFCs

To investigate whether cell density regulates the CD34 expression during passage of ECFCs, the cells from four individual donors were seeded on rat-tail collagen type I pre-coated six-well plates in PL-EGM at densities of 100, 500, 1000, and 2000 cells/cm^2^. The gene and surface expression of CD34 was evaluated by qRT-PCR and FC after 5 and 6 days of proliferation.

Proliferation assay was performed by seeding 100, 500, 1000, and 2000 cells/cm^2^ in 8 wells per seeding condition using 24-well plate. To enumerate the number of cells in cell cultures after 5 and 6 days of proliferation, the cells were fixed and stained with DAPI Vectashield Hardset (Vector Laboratories Ltd, BrunschwigChemie, the Netherlands). Image acquisition was performed using 4D-digital imaging microscope (DIM) by taking five images per well, and the cell number was calculated using ImageJ software.

### siRNA transfection and tube formation assay

To investigate the involvement of CD34 during sprout formation in fibrin matrices by PB-ECFCs, the cells were expanded for one passage in CMi to reduce the expression of CD34. After reaching confluence, the cells were expanded at 1:2 ratio and were incubated with ON-TARGET plus human CD34(947) siRNA-SMART pool (#L-019503-00-0005, Thermo Scientific, the Netherlands) for silencing CD34 (siRNA CD34 cells) or were incubated with ON-TARGETplus Non-targeting Pool siRNA (cat.no. D-001810-10-05,GE Dharmacon, Lafayette, CO) (siRNA NT cells). Non-transfected cells were used as control. Prior to transfection experiments, cells were starved for 4 h in M199 and were transfected using DharmaFECT4 reagent (Dharmacon). All siRNA and DharmaFECT4 were prepared in M199 + 10 % inactivated human serum supplemented with 10 ng/mL FGF-2 at final concentration of 20 nM. After 24 h of the transfection period the transfection medium was replaced by fresh PL-EGM medium. After additional 24 h the cells were detached and seeded on fibrin matrices as previously described. The transfection efficiency was evaluated by qRT-PCR and FC prior to seeding the cells on fibrin matrices. Sprout formation was initiated by stimulating the cells with combination of 10 ng/mL TNF-α and 10 ng/mL FGF-2 every day during 2-day period. Inhibition of tube formation was accomplished by transfecting the PB-ECFCs with siRNAs against uPA (Hs_PLAU_6 FlexiTube siRNA, cat.no. SI02662135, QiagenBenelux B.V., the Netherlands) which was prepared using the above described procedure. The tube formation ability of PB-ECFCs of four donors was determined in quadruplicate wells. Quantification of the length of formed tube-like structures was performed as previously described.

### siRNA transfection and endothelial barrier function assays

To investigate the involvement of CD34 in the maintenance of barrier function, the PB-ECFCs from three different donors were transfected with siRNA against CD34 or non-targeting siRNA as described in the previous section. Endothelial barrier function was evaluated with electric cell substrate impedance sensing (ECIS) as previously reported [34]. Briefly, the siRNA CD34 and siRNA NT cells were seeded in 1:1 density on gelatin-coated ECIS arrays, each containing 96 wells with 10 gold electrodes per well (96W10idf plates, Applied Biophysics, Troy, NY) in M199 + 10 % HSi + 10 ng/mL FGF-2. Resistance was measured at multiple frequencies to allow for calculation of resistance attributable to cell–cell adhesion (Rb) and to cell–matrix interaction (Alpha) over period of 24 h [34].

### Real-time polymerase chain reaction

The total RNA from PB-ECFCs was isolated using RNeasy MinElute Cleanup Kit (Qiagen, the Netherlands), and the RNA quality was tested with a NanoDrop 1000 spectrophotometer. Copy DNA (cDNA) was synthesized using the Cloned AMV first-strand cDNA synthesis kit from Invitrogen. The sequences of primers used for determination of genes of interest are given in Online resource Supp. Table 1.

Quantitative RT-PCR was performed using SYBR Green in an ABI 7500 sequence detection system (Applied Biosystems, Foster City, USA) and the following protocol: 2 min 50 °C, 10 min 95 °C and 40 cycles (15 s 95 °C, 1 min 60 °C) and dissociation curve. The relative expression levels of target genes were calculated with the housekeeping gene glyceraldehyde 3-phosphate dehydrogenase (GAPDH) by following equation as previous described [35]: Δ Ct sample = (Ct sample GENE) − (Ct sample HKG). The relative gene expression = 2 (Δ Ct sample 1 − Δ Ct Sample).

## Results

### Selection of CD34^+^ and CD34^−^ cells from PB-ECFCs

While PB-ECFC colonies arise from CD34^+^ cells [6], during subculturing both CD34^+^ and CD34^−^ ECFCs are encountered. To investigate whether CD34^+^-subcultured ECFCs are a better cell source for tissue engineering than their CD34^−^ counterparts, we compared the proliferation capacity of CD34^+^ with that of CD34^−^ cells as well as their sprouting ability in a 3D-fibrin matrix. Confluent cultures of PB-ECFCs from four donors were separated into CD34^+^ and CD34^−^ cells using CD34 magnetic beads. After each separation, the purity of yielded cell fraction was evaluated by flow cytometry (FC). Two sequential separations steps yielded highly purified CD34^+^ (95 % ± 0.8) and CD34^−^ (99 % ± 0.8) cell fractions (Online resource Supp. Figure 2).

**Fig. 1:**
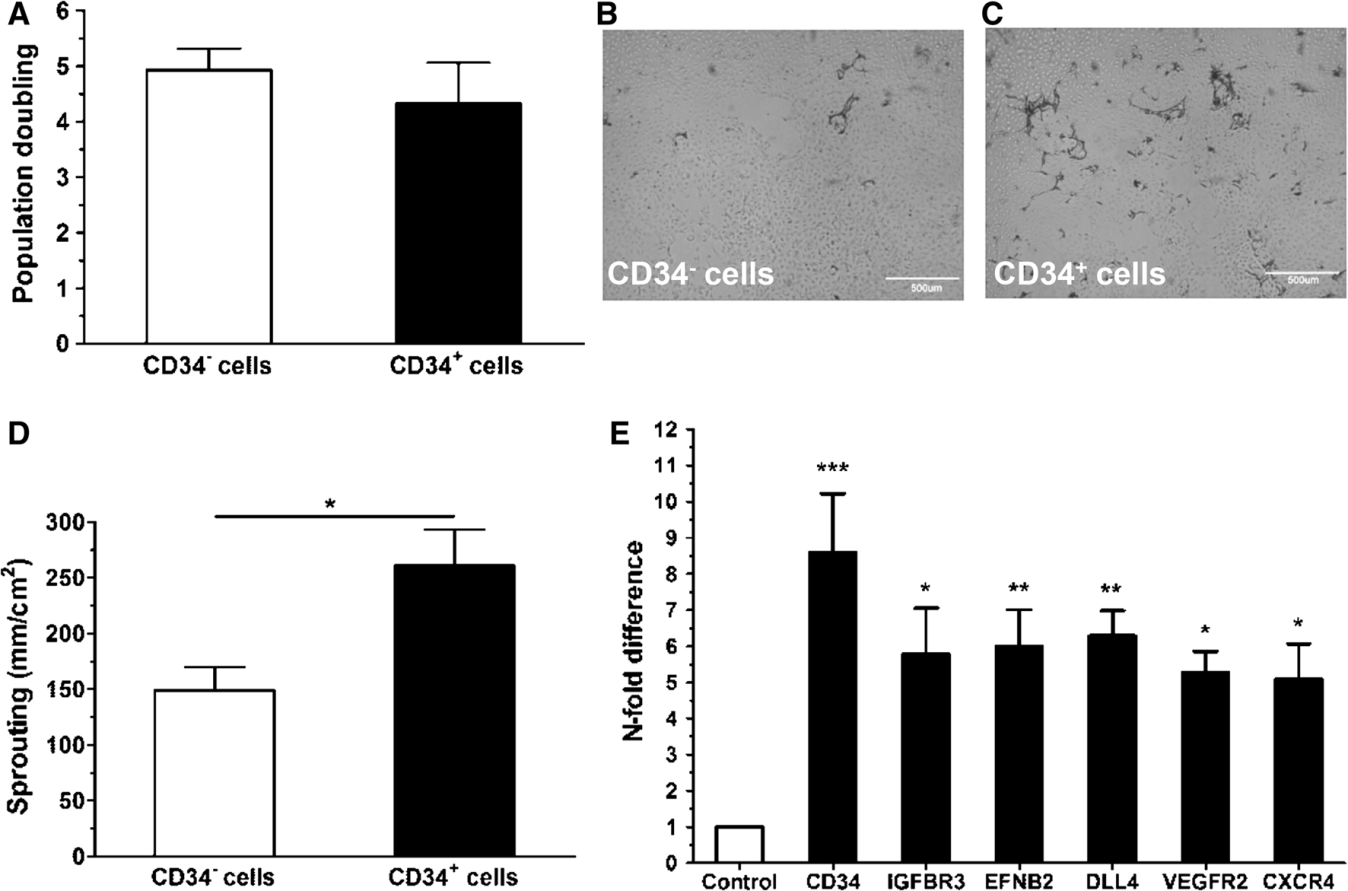
Angiogenic capacity of CD34^+^ PB-ECFCs. **a** Proliferation capacity of CD34^−^ (*open bar*) and CD34^+^ (*closed bar*) PB-ECFCs expressed as a mean ± SEM of population doubling. **b**–**c** Representative phase contrast images of sprout formation in fibrin matrices by CD34^−^ (**b**) and CD34^+^ (**c**) ECFCs stimulated with the combination of VEGF-A and FGF-2. **d** Quantification of sprouting ability of CD34^−^ (*open bar*) and CD34^+^ (*closed bar*) PB-ECFCs in fibrin matrices. Data are expressed as a mean ± SEM of mean length of formed sprouts of four independent experiments each performed with different donor. **e** mRNA levels of genes associated with tip-cell phenotype in CD34^+^ PB-ECFC compared to CD34^−^ cells. *Open bar* depict mRNA levels of tip-cell-associated genes in CD34^−^ cells. *Closed bars* represent mRNA levels of tip-cell associated genes in CD34^+^ cells

During the course of 7 days both cell fractions proliferated at same rate indicating that CD34^+^ ECFCs did not differ to CD34^−^ ECFCs with respect to the proliferation ability (Fig. 1a).

Stimulation with a combination of VEGF-A and FGF-2 induced sprout formation in fibrin matrices by both cell fractions (Fig. 1b, c). Comparison of sprouting ability of CD34^+^ and CD34^−^ cells revealed CD34^+^ ECFCs formed more sprouting structures than CD34^−^ ones (Fig. 1d). Previously we demonstrated that sprouting by ECFCs required u-PA and uPAR and is modulated by PAI-1 [30]. There were no significant differences observed between CD34^+^ and CD34^−^ ECFCs in the expressions of u-PA (1.1 ± 0.7), uPAR (1.1 ± 0.3), and PAI-1 (1.5 ± 0.7) (mean ± SEM, four different donors). Further phenotypical characterization of CD34^+^ ECFCs unveiled that CD34^+^ cells displayed an enrichment of genes usually accepted as markers of tip-cell phenotype (Fig. 1e). These data suggest that selection of CD34^+^ ECFCs might be feasible approach to facilitate the initiation of regenerative angiogenesis in ischemic tissues.

### CD34^+^ and CD34^−^ ECFCs are interchangeable phenotypes

As both CD34^+^ and CD34^−^ ECFCs originate from mononuclear cell fraction positive for CD34 [6], we wondered whether CD34^−^ ECFCs represent a separate lineage of cells or merely reflect a phenotype, which can re-acquire CD34 antigen. Therefore, we subsequently investigated whether the cells in culture that are negative for CD34 can re-express this protein on mRNA and protein level. Using magnetic beads the PB-ECFCs from four donors were separated on CD34^+^ and CD34^−^ ECFCs, and the efficiency of separation was evaluated by FC (Online resources Supp. Figure 2).

**Fig. 2:**
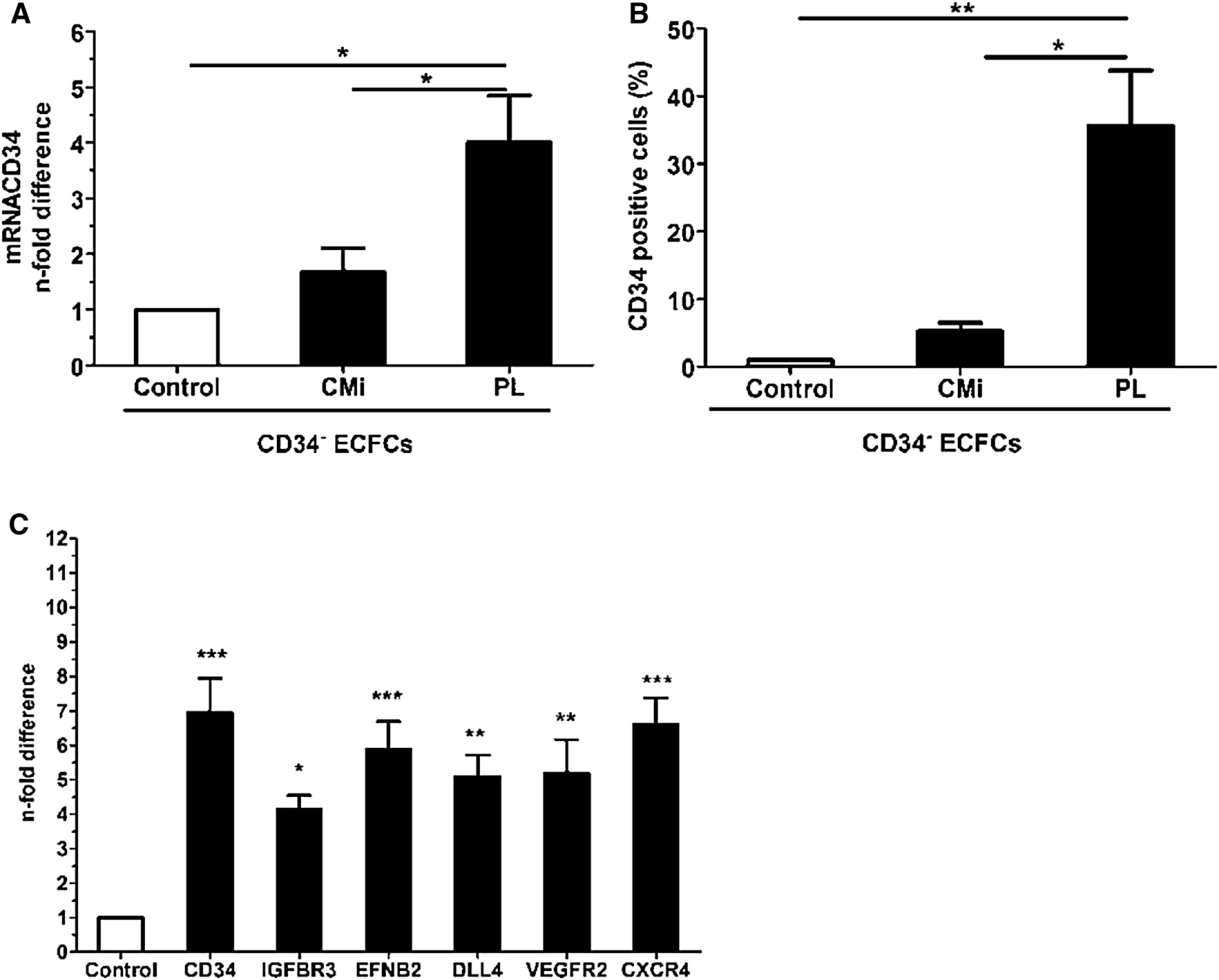
Expression of CD34 in CD34^+^ and CD34^−^ PB-ECFCs fractions after magnetic separation. **a** CD34 mRNA levels in CD34^−^ ECFCs after cell culture in PL-EGM or CMi. *Open bar* depicts the CD34 mRNA levels in CD34^−^ ECFCs obtained after separation and prior the seeding in PL-EGM or CMi. *Closed bars* represent CD34 mRNA levels in CD34^−^ CMi and CD34^−^ PL cells after 6 days in cell culture. **b** Flow cytometry evaluation of the number of CD34^+^ cells in CD34^−^ ECFCs cultures expanded in CMi or PL-EGM. Data are expressed as mean ± SEM percentage of cells positive for CD34 in the cell cultures expanded either in CMi or PL-EGM (*closed bars*). The control CD34^−^ cells are depicted with *open bar*. **c** mRNA levels of genes associated with tip-cell phenotype in CD34^+^ and CD34^−^ PB-ECFCs obtained after magnetic separation of CD34^−^ PL. *Open bar* depict mRNA levels of tip-cell associated genes in CD34^−^ cells, while *closed bars* represent mRNA levels of same genes in CD34^+^ cells

Both CD34^+^ and CD34^−^ fractions had comparable expressions of VE-cadherin, CD31, and VEGFR2 measured by FC, which were the same as in ECFCs before separation, which confirms the endothelial nature of both fractions (not shown). After separation, the CD34^−^ ECFCs were seeded and cultured in PL-EGM (CD34^−^ PL) or CMi (CD34^−^ CMi) and once the cells reached confluence (3–4 days), and the number of CD34^+^ cells was quantified by FC analysis, while in parallel the mRNA levels of CD34 were evaluated by qRT-PCR. The CD34^−^ PL ECFCs re-expressed significantly more CD34 than the control cells both at gene and at protein level (Fig. 2a, b).

Incubation of CD34^−^ cells in M199 medium supplemented with 3 % human serum albumin (HSA)for 24 h also induced upregulation of CD34 on the cell surface (Online resources Supp. Figure 3). This indicates that the expression of CD34 was reversible.

To investigate whether CD34^+^ ECFCs in the CD34^−^ fraction that re-expressed CD34 on cell surface are also characterized by the enrichment of tip-cell-associated genes (VEGFR2, DLL4, CXCR4, EFNB2, IGFBP3), we subsequently separated CD34^−^ PL that re-expressed CD34 into positive and negative for CD34 ECFCs and performed qRT-PCR. Indeed, CD34^+^ ECFCs obtained from CD34^−^ PL cells that re-expressed CD34 on cell surface also expressed significantly more mRNA transcripts of the genes related to tip-cell phenotype (Fig. 2c). This finding is in line with the data obtained from freshly separated cells (Fig. 1f).

Unexpectedly, the cells cultured in CMi exhibited only slight increases in CD34 mRNA and surface antigen levels compared to the initially seeded CD34^−^ cells (Fig. 2a, b). This suggests that serum components of the CMi do not favor the re-expression of CD34 on CD34^−^ ECFCs, indicating that the soluble factors present in serum play a role in regulation of CD34 in PB-ECFCs.

### Serum supplements affect CD34 expression in confluent ECFCs

**Fig. 3:**
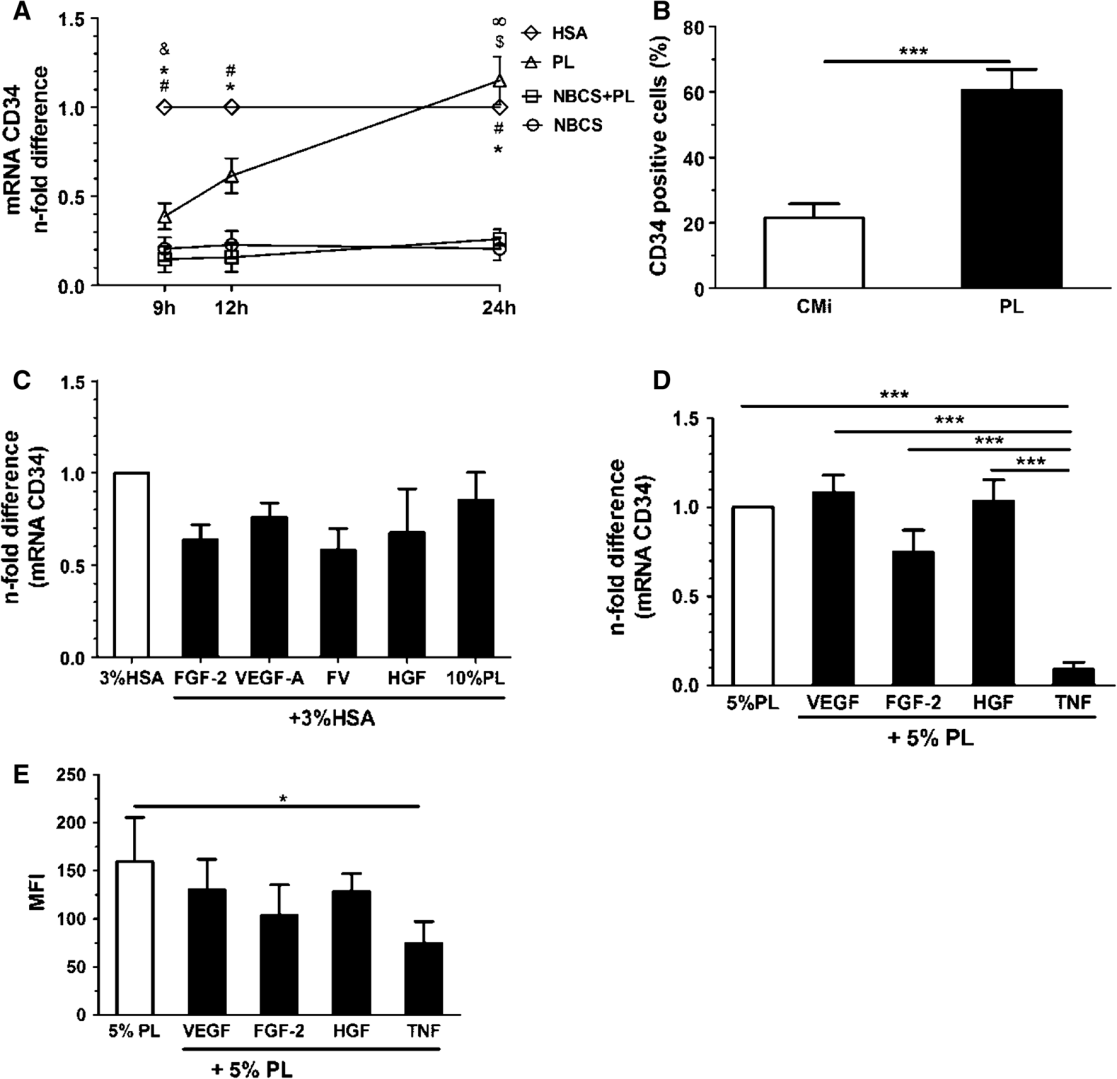
Effect of serum supplements on CD34 expression in PB-ECFCs. **a** Time course of CD34 induction in PB-ECFCs upon incubation with media supplemented with different serum supplements during 24-h period. *Open circle* depicts 10 % NBCS, *open square* depicts 10 % NBCS + 10 % PL, *open pointed up triangle* depicts 10 % PL, *open diamond* depicts 3 % HSA. Data are expressed as mean ± SEM of *n*-fold difference of CD34 mRNA levels compared to the mRNA levels in the cells incubated with M199 + 3 % HSA at each time point (HSA, *p* < 0.05: # to NBCS, * to NBCS + PL, & to PL; PL, *p* < 0.05: $ to NBCS, ∞ to NBCS + PL). **b** Flow cytometry assessment of CD34 expression in PB-ECFCs cultured in the presence of NBCS or PL. Data are expressed as a mean ± SEM percentage of cells positive for CD34 in the cell cultures incubated with CMi medium which contains 10 % NBCS (*open bar*) or M199 + 10 % PL (*closed bar*). **c** qRT-PCR evaluation of CD34 expression in cells incubated with VEGF-A (25 ng/mL), FGF-2 (10 ng/mL), FV (10 ng/mL FGF-2 + 25 ng/mL VEGF-A), HGF (10 ng/mL), and 10 % PL for 24 h prepared in M199 + 3 % HSA. Data are expressed as mean ± SEM of *n*-fold difference of CD34 gene levels in stimulated cells (*closed bars*) normalized to control cells incubated only in M199 + 3 % HSA (*open bar*). **d** qRT-PCR evaluation of CD34 expression in cells incubated with VEGF-A (25 ng/mL), FGF-2 (10 ng/mL), HGF (10 ng/mL), and TNF-α (10 ng/mL) for 24 h prepared in EBM-2 + 5 % PL. Data are expressed as mean ± SEM of *n*-fold difference of CD34 gene levels in stimulated cells (*closed bars*) normalized to control cells incubated only in EBM-2 + 5 % PL (*open bar*). **e** Flow cytometry evaluation of the effect of VEGF-A (25 ng/mL), FGF-2 (10 ng/mL), HGF (10 ng/mL), and TNF-α (10 ng/mL) for 24 h prepared in EBM-2 + 5 % PL on CD34 expression in PB-ECFCs. *Closed bars* represent the effect of each of soluble factors prepared in control medium (*open bar*) on CD34 expression. Data are expressed as mean ± SEM of mean fluorescence intensity (MFI) of CD34 antibody fluorescence intensity minus autofluorescence of matched isotype antibody

We subsequently evaluated which serum supplement(s) might modulate CD34 expression in PB-ECFCs. To that end we compared platelet lysate and serum used in CMi, in particular 10 % NBCS and 10 % HS. Confluent monolayers of PB-ECFCs grown in CMi were transferred into M199 medium supplemented with 10 % NBCS, 10 % PL, 10 % NBCS/10 % PL, or 3 % HSA and incubated for 9, 12, and 24 h. In the presence of only 3 % HSA, CD34 mRNA expression increased fivefold within 9 h and remained constant thereafter (Fig. 3a).

In the presence of PL a slow increase was observed reaching two- to sixfold after 9 and 24 h, respectively. However, in the presence of NBCS—a serum component of CMi, the CD34 mRNA expression remained at the original low level. Also when NBCS and PL were combined the CD34 mRNA expression remained low (Fig. 3a). Apparently, NBCS did not stimulate CD34 expression and had a dominant suppressive effect over PL as well. The difference between exposure to medium containing NBCS such as CMi and that to PL was also reflected in the cell surface expression of CD34 antigen (Fig. 3b).

To evaluate whether pro-angiogenic growth factors and cytokines might affect the CD34 expression in confluent PB-ECFCs, we compared their effects both in PB-ECFCs that were incubated in M-199 supplemented with 3 % HSA as well as EBM-2 + 5 % PL. The growth factors FGF-2, VEGF-A, and HGF did not change CD34 mRNA or surface antigen expression significantly in both conditions (Fig. 2c–e). Addition of 5–10 % PL to HSA-containing medium also did not alter CD34 mRNA expression. The inflammatory cytokine TNF-α reduced the mRNA and antigen expression significantly (Fig. 2d, e), in agreement with previous finding on endothelial cells [13].

### Proliferation and seeding density alter CD34 expression in ECFCs

**Fig. 4:**
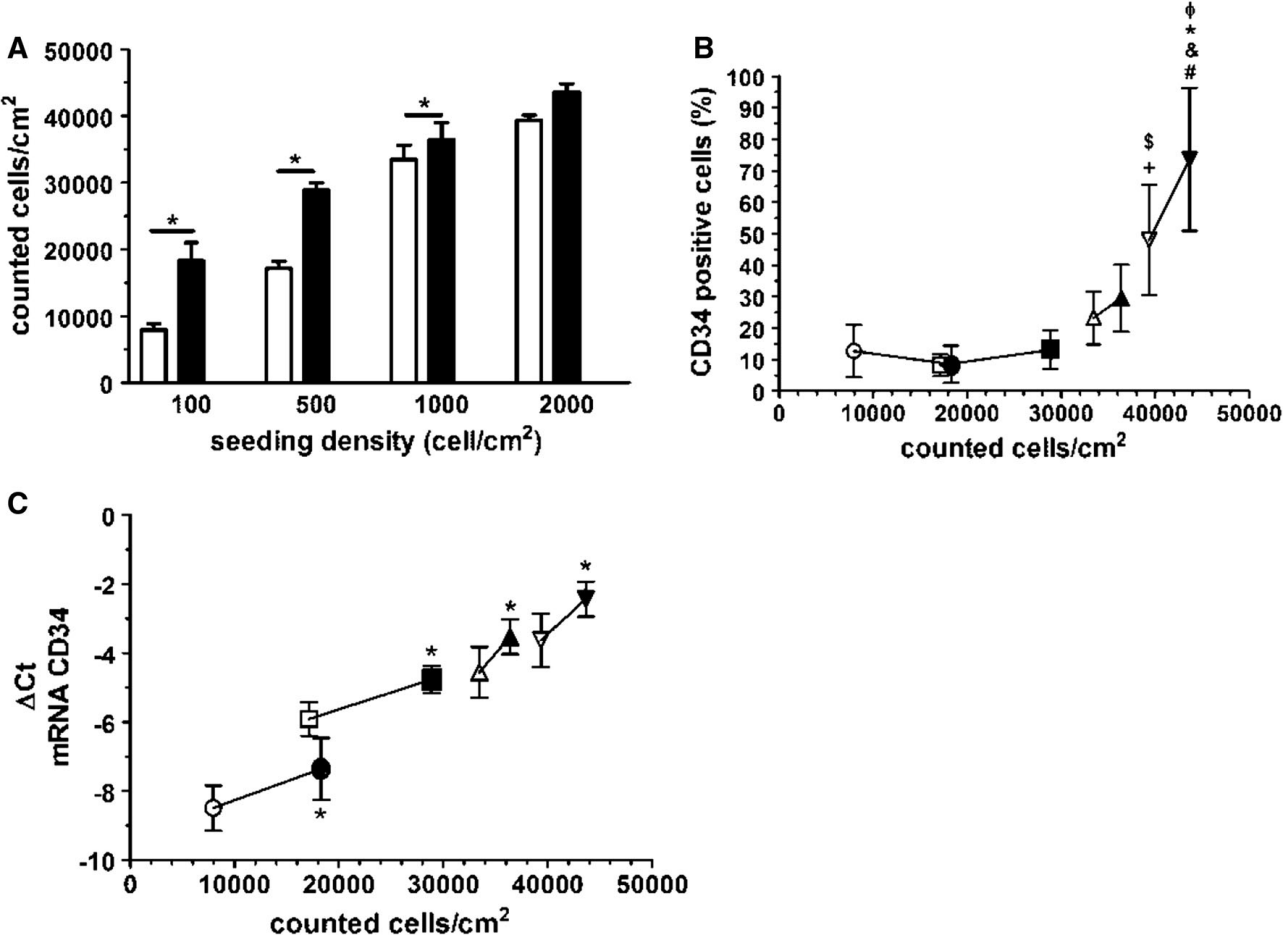
Effect of cell density and proliferation on CD34 expression in PB-ECFCs. **a** Proliferation ability of PB-ECFCs seeded at different cell densities after five (D5, *open bars*) and six (D6, *closed bars*) days. Results represent mean ± SEM of counted cells/cm^2^ from four independent experiments each performed with different donor. **b** Relation of CD34 surface expression and proliferation in PB-ECFCs seeded at different cells densities. Flow cytometry evaluation of the number of CD34^+^ cells in cell cultures established with seeding PB-ECFCs at different densities after five (D5, *open symbols*) and six (D6, *closed symbols*) days of proliferation. Data represents the percentage of cells positive for CD34 in the cell cultures established by seeding 100 (*open circle*), 500 (*open square*), 1000 (*open pointed up triangle*), and 2000 (*open pointed down triangle*) cells/cm^2^ after 5 (*open symbols*) and 6 (*closed symbols*) days proliferation plotted against the number of cells counted at the end of proliferation. (*p < 0.05). ^$^indicates *p* < 0.05 to 100 cells/cm^2^ at D5, + indicates *p* < 0.05 to 500 cells/cm^2^ at D5; #, *, & indicate *p* < 0.05 to 100, 500, and 1000 cells/cm^2^ at D6; Φ indicates *p* < 0.05 to 2000 cells/cm^2^ at D5. **c** Relation of CD34 gene expression and proliferation in PB-ECFCs seeded at different cells densities. Comparison of mRNA levels of CD34 at day 5 (D5, *open symbols*) and day 6 (D6, *closed symbols*) in cell cultures established by seeding 100 (*open circle*), 500 (*open square*), 1000 (*open pointed up triangle*), and 2000 (*open pointed down triangle*) cells/cm^2^ of PB-ECFCs plotted to the number of cells counted at the end of proliferation. Data represent the mean ± SEM of delta *Ct* values of CD34 mRNA levels at day 6 (D6, *closed symbols*) compared to delta Ct of CD34 mRNA levels at day 5 (D5, *open symbols*) which served as control

To investigate whether cell density regulates CD34 expression during passage of ECFCs, the cells from four individual donors were seeded on rat-tail collagen type I in PL-EGM at densities of 100, 500, 1000, and 2000 cells/cm^2^. The CD34 expression was evaluated after five (D5) and 6 days (D6) of culturing using PCR and FC. During the course of proliferation assay, the conditions with 100, 500, and 1000 cells/cm^2^ contained significantly more cells at D6 compared to D5, indicating that the cells continued to proliferate in 24-h period between the two time points reaching different levels of confluence (Fig. 4a, Online resources Supp. Figure 4a–f).

On contrary, at 2000 cells/cm^2^ at D5 and D6 similar numbers of cells were counted suggesting that the cell cultures were confluent and the cells had stopped dividing (Fig. 4a, Online resources Supp. Figure 4g–h). Before the start of experiment the percentage of CD34^+^ cells in the cultures obtained from different donors was 83.3 ± 11.5 % as measured by FC. Strong reduction in the number of CD34^+^ cells was observed in the cultures established with 100, 500, and 1000 cells/cm^2^ at D5 and D6, while a cell-density-dependent restoration of CD34 content occurred as most clear in 2000 cells/cm^2^ cultures (Fig. 4b). These data indicate that proliferation as well as cell density affects the regulation of CD34 in ECFCs probably through establishment of cell–cell contacts. Progressive increase in CD34 mRNA levels between D5 and D6 was also observed in all seeding conditions as measured by qRT-PCR (Fig. 4c), suggesting that establishment of cell–cell contact also promotes CD34 transcription irrespectively of proliferation status of cell cultures.

### CD34 alters barrier function of PB-ECFCs

Previous studies reported that CD34 molecules on two adjacent EC cells play an important role in initiation of lumen formation in mouse embryo by generation of repulsive forces that destabilized cell–cell contacts [36]. Considering that disassembling of the continuity of EC monolayer is one of the first steps in angiogenic sprouting, we investigate whether silencing of CD34 changes the barrier properties of ECFC. PB-ECFCs from three donors were transfected with siRNA targeting CD34. Efficient silencing of CD34 at the cell surface and gene level was confirmed by FC and qRT-PCR (Online resources Supp. Figure 5).

**Fig. 5:**
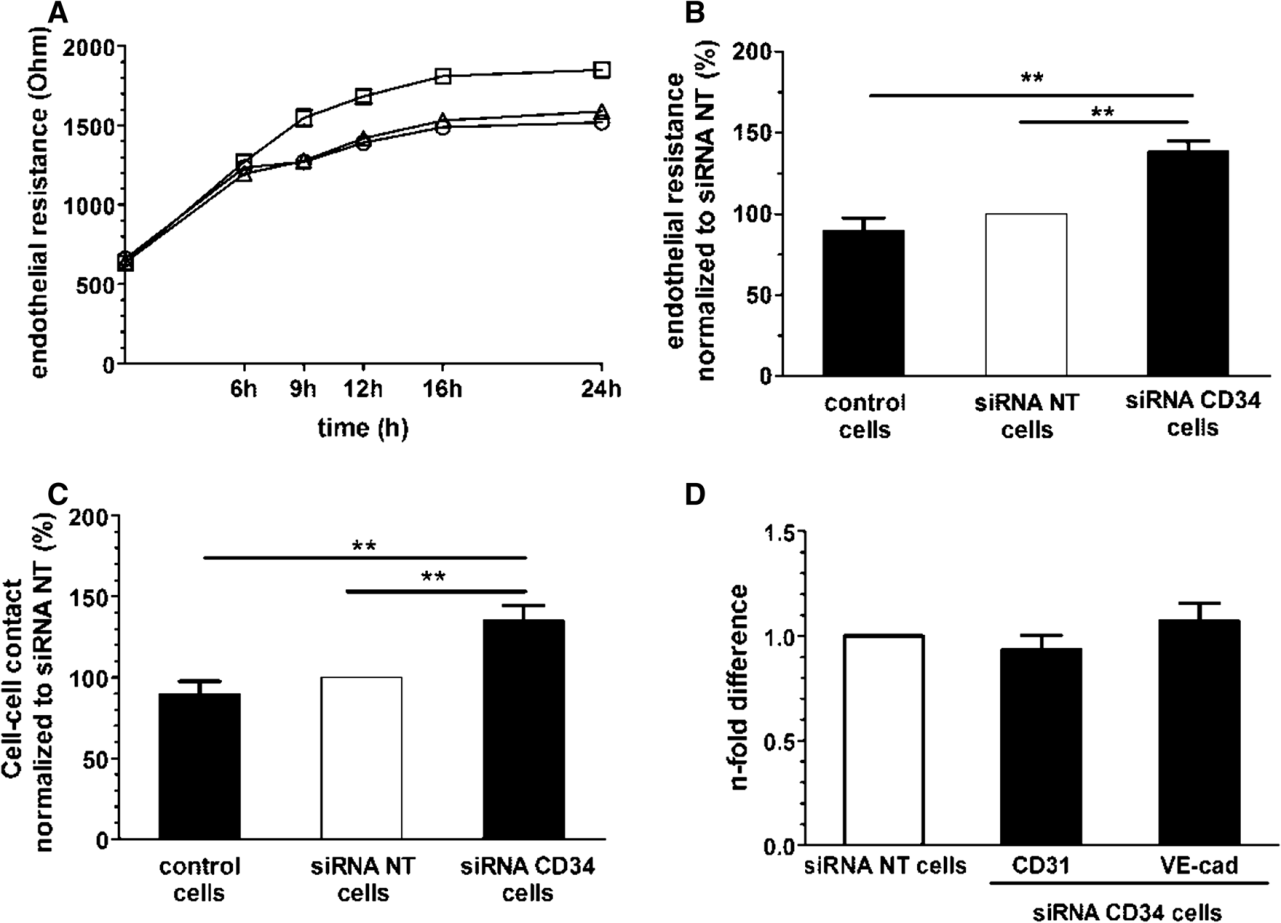
Role of CD34 during maintenance of barrier function in PB-ECFCs. **a** Barrier function of control PB-ECFCs (*open circle*), cells transfected with non-targeting siRNA (*open pointed up triangle*), and cells with CD34 silenced by siRNA (*open square*) over period of 24 h. Data are expressed as absolute endothelial resistance (Ohm). **b** Absolute endothelial resistance of confluent monolayers of PB-ECFCs of three independent experiments each performed with different donor. Data of control and siRNA CD34 cells (*closed bars*) are normalized to the values of cells transfected with non-targeting siRNA (*open bars*) which served as control. **c** Strength of cell-cell interaction in confluent monolayers of control cells, cells transfected with non-targeting siRNA and cells transfected with siRNA CD34. Data of control and siRNA CD34 cells (*closed bars*) are normalized to the values of cells transfected with non-targeting siRNA (*open bars*) which served as control. **d** CD31 and VE-cadherin mRNA levels in PB-ECFCs transfected with siRNA CD34. Results represent the mean ± SEM of four independent experiments each performed with different donor. *Open bar* depicts the CD34 mRNA levels in cells transfected with non-targeting siRNA. *Closed bars* represent the mRNA levels of CD31 and VE-cadherin in the cells transfected with siRNA CD34

The transfection procedure did not alter the gene expression levels of CD31 and VE-cadherin, which play an important role in cell–cell interaction (Fig. 5d).

ECIS measurement over period of 24 h revealed that silencing of CD34 caused an increase of total resistance of the ECFC monolayer (Fig. 5a). Quantification at the end of 24-h period confirmed that a significantly higher resistance was observed in the cell monolayers with silenced CD34 than in the controls, non-transfected cells or the cells transfected with non-targeting siRNA (Fig. 5b). After computational modeling of the endothelial electrical resistance which enables discrimination between cell–cell and cell–matrix interactions, we detected that the increase in total endothelial resistance upon silencing of CD34 was attributable to increased cell–cell interactions (Fig. 5c). This indicates that CD34 favors the loosening of cell–cell interaction between PB-ECFCs.

### Silencing of CD34 reduces sprout formation ability of PB-ECFCs

To further evaluate the contribution of CD34 to sprouting, we assessed the impact of silencing of CD34 expression by siRNA on sprout formation in fibrin matrices. PB-ECFCs transfected with siRNA CD34 (siRNA CD34 cells), or non-targeting siRNA pool (siRNA NT cells) as well as control non-transfected ECFCs were seeded on fibrin matrices and simultaneously stimulated with TNF-α and FGF-2 (TF). siRNA CD34 significantly reduced the mRNA and surface expression of CD34 as validated by qRT-PCR and FC (Online resources Supp. Figure 5).

**Fig. 6:**
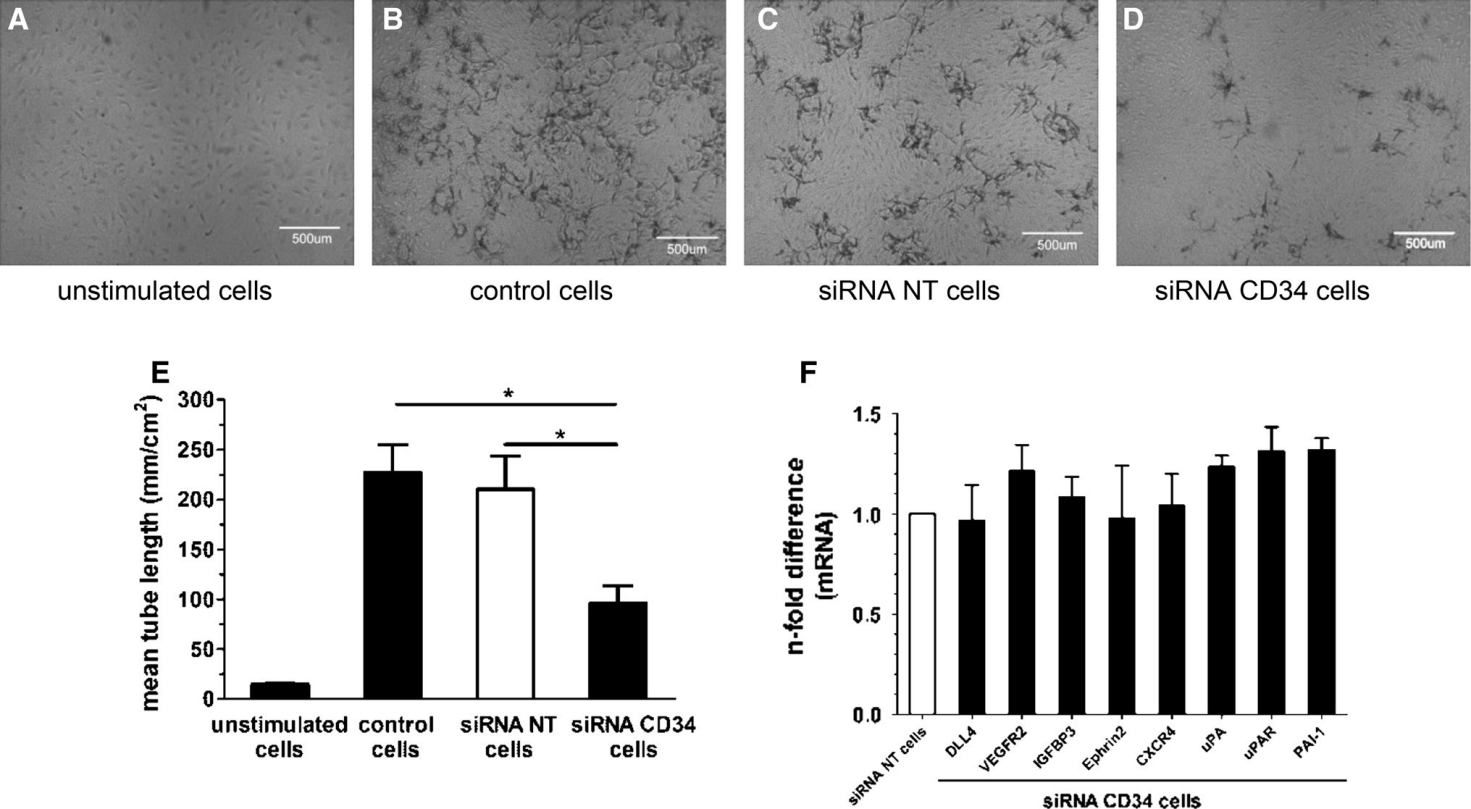
CD34 modulates sprouting response of PB-ECFCs in fibrin matrices. **a-d** Representative phase contrast pictures of sprout formation in fibrin matrices. **a** unstimulated cells; **b** control, non-transfected cells; **c** cells transfected with non-targeting siRNA; **d** cells transfected with CD34 siRNA. **e** Effect of silencing of CD34 in PB-ECFCs on sprout formation in fibrin matrices. Data are expressed as a mean ± SEM of mean length of formed sprouts of four independent experiments each performed with different donor. **f** mRNA levels of tip-cell-associated and fibrinolytic genes in PB-ECFCs after silencing of CD34. Data are expressed as mean ± SEM of *n*-fold difference of mRNA levels of depicted genes in the siRNA CD34 transfected cells (*closed bars*) compared to the cells transfected with non-targeting siRNA (*open bars*) which served as control

During the course of 2-day stimulation period, the siRNA NT cells exhibited similar sprouting response as the control, non-transfected cells indicating that the transfection procedure did not alter the sprouting response of PB-ECFCs (Fig. 6a–c), while the siRNA CD34-transfected cells exhibited significantly reduced sprout formation (Fig. 6d, e).

In line with an involvement of the fibrinolytic system in sprouting-associated pericellular proteolysis, the siRNA u-PA-treated cells completely failed to form sprouting structures in fibrin matrices sprout formation (Online resources Supp. Figure 6).

To investigate whether the observed results are related to the difference in expression of genes associated with tip-cell phenotype or involved in pericellular proteolysis accompanying tube formation in fibrin matrices, qRT-PCR was performed to compare the mRNA levels of set of genes in siRNA CD34, siRNA NT, and control cells. Silencing of CD34 in ECFCs did not alter the mRNA levels of tip-cell-associated genes, while uPA and PAI-1 were moderate increased in siRNA CD34 cells compared to siRNA NT cells (Fig. 6f). Therefore, the sprouting response of siRNA CD34 cells showed that the CD34 protein might indeed play a role during invasion of fibrin matrices, but its engagement during the sprouting was independent of the induction of tip-cell-associated genes in ECFCs.

## Discussion

Findings in this study demonstrated that compact colonies of ECFCs derived from the mononuclear cell fraction of peripheral blood develop hybrid cellular expression of CD34, i.e., contain CD34^+^ and CD34^−^ cells, which both express endothelial cell-specific markers. These CD34^+^ and CD34^−^ ECFCs are not a separate cell lineages but merely reflect a different but interchangeable phenotype of endothelial cells. Our data revealed that CD34 is inducible in the CD34^−^ ECFCs and its regulation depends on cell density, and serum supplements. Functional assays and phenotypical characterization of CD34^+^ and CD34^−^ ECFCs demonstrated that CD34^+^ cells are characterized by higher sprouting capacity in fibrin matrices and enrichment of tip-cell-related genes compared to their CD34^−^ counterparts. Silencing of CD34 by siRNA caused improvement in the barrier function of ECFCs’ monolayers and reduced the extent of the tubular structures formation by ECFCs, suggesting a causal role for CD34, but did not affect the expression of tip-cell-associated genes.

### CD34 (re)expression during culture of ECFCs

The re-expression pattern of CD34 in CD34^−^ ECFCs was in agreement with previous reports on the induction of CD34 in human umbilical vein endothelial cells (HUVEC) [22] and hematopoietic stem cells [37]. We observed an increase in CD34 expression in confluent cells, similar as but at a much higher level than previously observed in HUVEC. Furthermore, the present study shows that CD34 was repressed in medium supplemented with NBCS and human serum, and became re-expressed after exposure of ECFCs to medium supplemented with human serum albumin or platelet lysate. Beside by serum, the CD34 expression in ECs might be regulated by pro-angiogenic factors or inflammatory cytokines [13, 34]. Although the previous reports suggested that FGF-2 and VEGF-A reduced or increased the CD34 expression in dermal microvascular ECs (DMVEC) or HUVEC, respectively [22, 34], both angiogenic factors as well as HGF did not alter the CD34 expression in PB-ECFCs cultured in the presence of either 3 % HSA, 5 % PL or NBCS. This may be due to the strong suppressive action of NBCS and the much higher CD34 levels that are reached in platelet lysate-exposed ECFCs than in primary cultures of HUVEC and DMVEC. Indeed, according to Barclay et al. [39] ECFCs belong to the endothelial lineage yet they differ in their response to environmental conditions. Alternatively, the effect of growth factors may influence growth rate in endothelial cells, which it is already very high in ECFCs. However, Hellwig et al. [38] reported that CD34 regulation is independent from the induction of proliferation. Furthermore, the state of confluence affects CD34 expression in ECFCs (this study) as well as in other primary endothelial cells [13, 34].

### Increased sprouting of CD34^+^ ECFCs in fibrin matrices

Functional assessment of sprouting of CD34^+^ and CD34^−^ separated cells showed that combined use of FGF-2 and VEGF induced more spout formation in the fibrin matrices seeded with CD34^+^ ECFCs compared to cells that lack this molecule, while both cell fractions proliferated at same rate. Silencing of CD34 in mixed CD34^+^/CD34^−^ ECFCs cultures also reduced formation of endothelial sprouts, suggesting a causal role of CD34 in enhanced tube formation. Increased sprouting was not related to differences in fibrinolytic gene profile of CD34^+^ and CD34^−^ fractions since both cell subsets have comparable basal mRNA levels of uPA, uPAR, PAI-1, t-PA, and MMP-14. Enrichment of tip-cell-related genes in CD34^+^ ECFCs might underlie increased sprout formation in fibrin matrices due to the increased mRNA levels of VEGFR2 or CXCR4, which are important regulators of the sprouting phase of angiogenesis [36, 37]. In addition, the CD34 re-expression in separated CD34^−^ cells was accompanied with increased levels of tip-cell-related genes suggesting that CD34 can be used as a marker for selection of ECFCs with distinct phenotype compared to CD34^−^ selected cells.

Recently Ferreras et al. [31] reported on two separate types of ECFC colonies, one CD34^+^ with densely growing cells and a second CD34^−^ type that grows in a more dispersed way. These authors concluded that only the CD34^−^ cells were capable of sprouting when embedded in fibrin or seeded onto Matrigel, while CD34^+^ were not. In contrast to Ferreras’s report [31], here presented data show that CD34^+^ ECFCs are able to form tubular endothelial structures in a fibrin matrix and to an larger extent than CD34^−^ cells. Furthermore, our data show that CD34^+^ and CD34^−^ represent both distinct endothelial but interchangeable phenotypes rather than separate cell lineages in a mixed population of ECFCs. Our studies do not exclude the existence of another CD34^−^ population of sparsely growing late outgrowth cells. We did observe such colonies in our initial cultures, but these colonies had a poor propagation and could not be cultured further than passage 4 in our experimental conditions.

It is not excluded that in vitro expansion in platelet lysate might accentuated the angiogenic phenotype of CD34^+^ ECFCs. This cell fraction was not only able to promote sprout formation more than CD34^−^ selected ECFCs, but also displayed a higher expression of tip-cell-associated genes. However, as silencing of CD34 did not affect the expression of tip-cell-associated genes, another mediator probably controls both CD34 and the other genes. Our observations on tip-cell gene expression by CD34^+^ ECFCs are in line with observations of Siemerink et al. [22] on CD34^+^ HUVEC. However, opposite to the observations of these latter authors, both CD34^+^ and CD34^−^ ECFCs proliferated in our conditions at similar rates.

### CD34 is involved in regulation of barrier function in PB-ECFCs

Our observation that CD34 is increased in fully confluent monolayers of PB-ECFCs and the accumulation of CD34 antigen staining at the cell margins suggests the involvement of this molecule in modulating cell–cell contacts. Preservation of intact cell–cell contact is an indispensable component of the barrier function of endothelial monolayer. However, silencing of CD34 induced increase in the strength of cell–cell interaction leading to augmentation of the barrier function, i.e., reduced permeability, of monolayers of PB-ECFCs. The reduction in endothelial barrier function by CD34 may seem counterintuitive as CD34 is increased in confluent endothelial cells. However, a similar effect was observed during mouse embryogenesis where the negatively charged CD34 molecules induced local repulsion of cell contacts between sprouting ECs thus enabling the formation of the lumen of future blood vessel [36]. Furthermore, in postnatal life, CD34 is expressed by EC lining the microvasculature in vivo [18]. This antigen, beside on the cell surface, is also distributed within the contact area between adjacent endothelial cells especially on the interdigitating invaginations/processes located close to the luminal side while the tight junctions appear devoid of CD34 [18]. Residing at the entry of tight junctions the CD34 might play a role in the first steps of opening the cell–cell contact area or in preventing closure of junctions once they open. During angiogenesis, it might be helpful for the EC to highly express CD34 on cell surface in order to reduce the stability of endothelial monolayer thus ensuring favorable conditions for the initiation of sprouting. Data obtained from sprouting assay with silencing of CD34 by siRNA support this hypothesis.

### CD34 and tip-cell gene expression

CD34^+^ ECFCs formed more sprouts and exhibited higher mRNA levels of genes associated with tip-cell phenotype than CD34^−^ ECFCs. This suggests that CD34 in concordance with increased expression of tip-cell genes might be involved during sprout formation by ECFCs. However, the silencing of CD34 experiment has shown that—while CD34 positivity was accompanied by increased expression of tip cells, the deletion of CD34 by itself was not sufficient to reduce the expression of these tip-cell genes. This indicates that CD34 has no causal role in enhancing the expression of these tip-cell genes in PB-ECFCs. Apparently, while selecting CD34^+^ cells, we also selected for a hierarchically higher regulator that enhances both CD34 and tip-cell gene expression. This common gene or transcription factor that drives this process in PB-ECFCs is presently still unknown.

### CD34^+^ versus CD34^−^ ECFCs

An interesting observation on CD34 hybrid ECFCs was made by Lee et al. [40], who suggested that the CD34 hybrid colonies contained both CD34^+^ ECFCs and CD34^−^ ECFCs, the latter acting as niche-supporting cells. The existence of niche-supporting cells fits well within current ideas of maintenance of stemness [41]. As we obtained 95 % CD34^+^ cells by cell separation, it is difficult to rule out fully the possibility that potential niche-supporting cells escaped the separation. However, we did not observe a difference in the proliferative capacity of CD34^+^ and CD34^−^ cells, and after a while both populations finally regressed to the same ratio CD34^+^/CD34^−^ cells, a ratio that was mainly influenced by cell density and culture conditions. The possibility that CD34^+^ ECFCs can acquire niche-supporting properties themselves, or that niche-supporting cells may express temporarily CD34 needs further attention.

## Conclusion

This manuscript demonstrates the plasticity and regulation of CD34 expression in ECFCs cultured from adult human peripheral blood, as well as the stimulatory role of this molecule on endothelial tube formation in a 3D-fibrin matrix. It also pointed to a contribution of CD34 to local barrier destabilization that may accompany sprouting. While deletion of CD34 expression by siRNA demonstrates a causal role of CD34 in improved tube formation—independent of the regulation of several important tip-cell genes, selection of CD34^+^ cells from CD34^+^/CD34^−^ hybrid ECFC cultures will only have effect when separation occurs immediately before application of the CD34^+^ cells. After several days the original hybrid expression pattern of CD34 will be regained by the subcultured PB-ECFCs. Notwithstanding, this mixed phenotype still displays the same sprouting ability and proliferation rate as the purified CD34^+^ ECFCs.

## Electronic supplementary material

Below is the link to the electronic supplementary material.

Supplementary material 1 (PDF 7206 kb)

## Abbreviations

TNF-α: Tumor necrosis factor-alpha
FGF-2: Fibroblast growth factor-2
VEGF-A: Vascular endothelial growth factor-A
HS: Human serum
NBCS: Newborn calf serum
HSA: Human serum albumin
HGF: Hepatocyte growth factor
ECIS: Electric cell substrate impedance sensing
GAPDH: Glyceraldehyde 3-phosphate dehydrogenase
uPA: Urokinase plasminogen activator
uPAR: Urokinase plasminogen activator receptor
tPA: Tissue plasminogen activator
PAI-1: Plasminogen activator inhibitor-1
VEGFR-2: Vascular endothelial growth factor receptor-2
DLL4: Delta-like ligand 4
IGFBP3: Insulin growth factor-binding protein 3
EFNB2: Ephrin-B2
MMP-14: Matrix metaloproteinases-14
