# Supplementary material for: CD34 expression modulates tube-forming capacity and barrier properties of peripheral blood-derived endothelial colony-forming cells (ECFCs)
Source: Angiogenesis. 2016 Apr 4;19:325–38. doi: 10.1007/s10456-016-9506-9 (PMC4930476; doi:10.1007/s10456-016-9506-9)
Supplement: Supplementary file 1 — Supplementary material 1 (PDF 7206 kb) [file 10456_2016_9506_MOESM1_ESM.pdf]

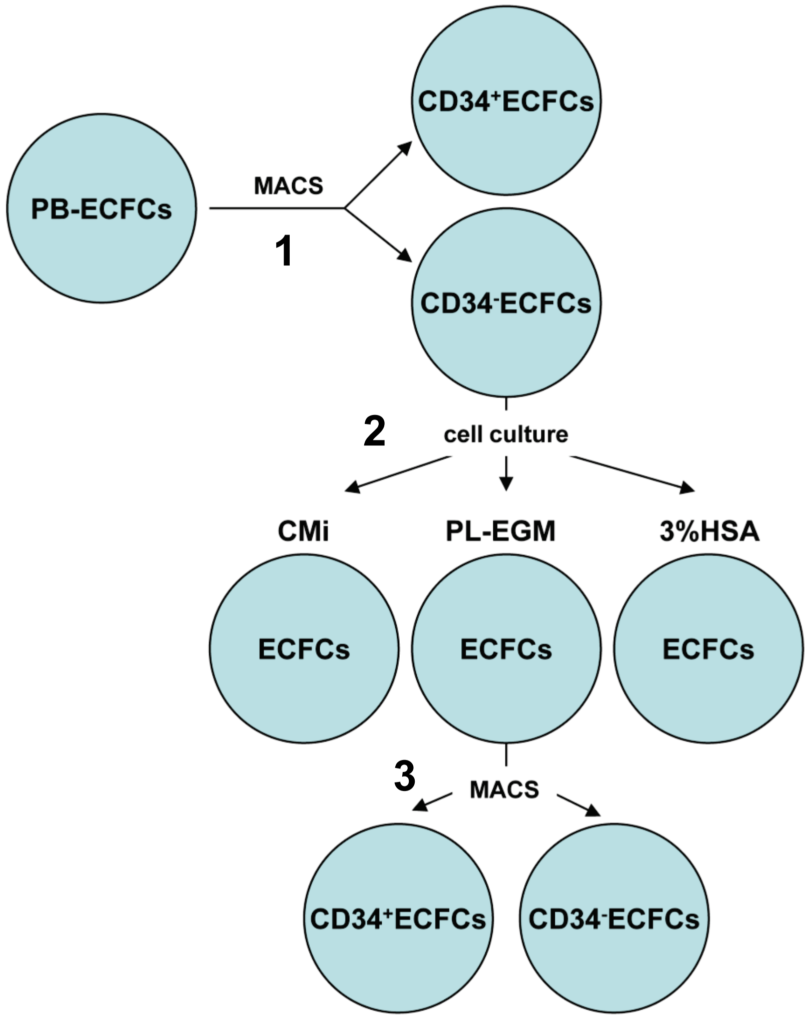

Supp. Fig.1 Schematic overview of experimental layout of CD34 separation and re-expression

1) PB-ECFCs cell cultures were separated on $CD34^+$ and $CD34^-$ cells by MACS.

2) Next, the $CD34^-$ cells were further cultured in CMi and PL-EGM until confluence. $CD34^-$ cells were also incubated in M199 supplemented with 3%HSA for 24h.

3) Second MACS step was used to obtain $CD34^+$ and $CD34^-$ cells from $CD34^-$ cells that were cultured in PL-EGM. Subsequently, the obtained $CD34^+$ and $CD34^-$ ECFCs were used for experimental purposes.

Supplementary table 1. List of primers.

Gene	Forward primer	Reverse primer
GAPDH[1]	GGTCTCCTCTGACTTCAACA	AGCCAAATTCGTTGTCATAC
CD34[2]	ACCACTAGCACTAGCCTTGC	CCTTCTTAAACTCCGCACAGC
uPA [3]	ACTACTACGGCTCTGAAGTCACCA	GAAGTGTGAGACTCTCGTGTAGAC
uPAR[4]	CATGCAGTGTAAGACCAACGGGGA	AATAGGTGACAGCCCGGCCAGAGT
tPA[3]	CCAGATCGAGACTCAAAGCC	GACCCATTCCCAAAGTAGCA
PAI-1[3]	GCACAACCCCACAGGAAC	TGCTTCAAACTTCTCTCCCAG
VEGFR-2[5]	TGGGAACCGGAACCTCACTATC	GTCTTTTCCTGGGCACCTTCTATT
DLL4[6]	GACCACTTCGGCCACTATGT	CCTGTCCACTTTCTTCTCGC
CXCR4	ACC ATGGAGGGGATCAGTATATACA	ACAGGGTTCCTTCATGGAGTCA
IGFBP3[7]	CCATGACTGAGGAAAGGAGCTC	TGCAGCAGGGCAGAGTCTC
EFNB2	GAAAATACCCCTCTCCTCAACT	CTTCGGAACCGAGGATGTTGTTC
MMP14[8]	GCAGAAGTTTTACGGCTTGCAA	CCTTCGAACATTGGCCTTGAT

1. Brooks, S. P., Trueman, R. C. & Dunnett, S. B. Striatal lesions in the mouse disrupt acquisition and retention, but not implicit learning, in the SILT procedural motor learning task. *Brain Res.* **1185,** 179–188 (2007).

2. Brunet De La Grange, P. *et al.* Oxygen concentration influences mRNA processing and expression of the cd34 gene. *J. Cell. Biochem.* **97,** 135–144 (2006).

3. Houard, X. *et al.* Topology of the fibrinolytic system within the mural thrombus of human abdominal aortic aneurysms. *J. Pathol.* **212,** 20–28 (2007).

4. Li, Y. & Sarkar, F. H. Down-regulation of invasion and angiogenesis-related genes identified by cDNA microarray analysis of PC3 prostate cancer cells treated with genistein. *Cancer Lett.* **186,** 157–164 (2002).

5. Smadja, D. M. *et al.* PAR-1 activation on human late endothelial progenitor cells enhances angiogenesis in vitro with upregulation of the SDF-1/CXCR4 system. *Arterioscler. Thromb. Vasc. Biol.* **25,** 2321–2327 (2005).

6. Williams, C. K., Li, J.-L., Murga, M., Harris, A. L. & Tosato, G. Up-regulation of the Notch ligand Delta-like 4 inhibits VEGF-induced endothelial cell function. *Blood* **107,** 931–939 (2005).

7. Takaoka, M. *et al.* Epidermal Growth Factor Receptor Regulates Aberrant Expression of Insulin-Like Growth Factor-Binding Protein 3. *Cancer Res.* **64 ,** 7711–7723 (2004).

8. Munoz-Najar, U. M., Neurath, K. M., Vumbaca, F. & Claffey, K. P. Hypoxia stimulates breast carcinoma cell invasion through MT1-MMP and MMP-2 activation. *Oncogene* **25,** 2379–2392 (2005).

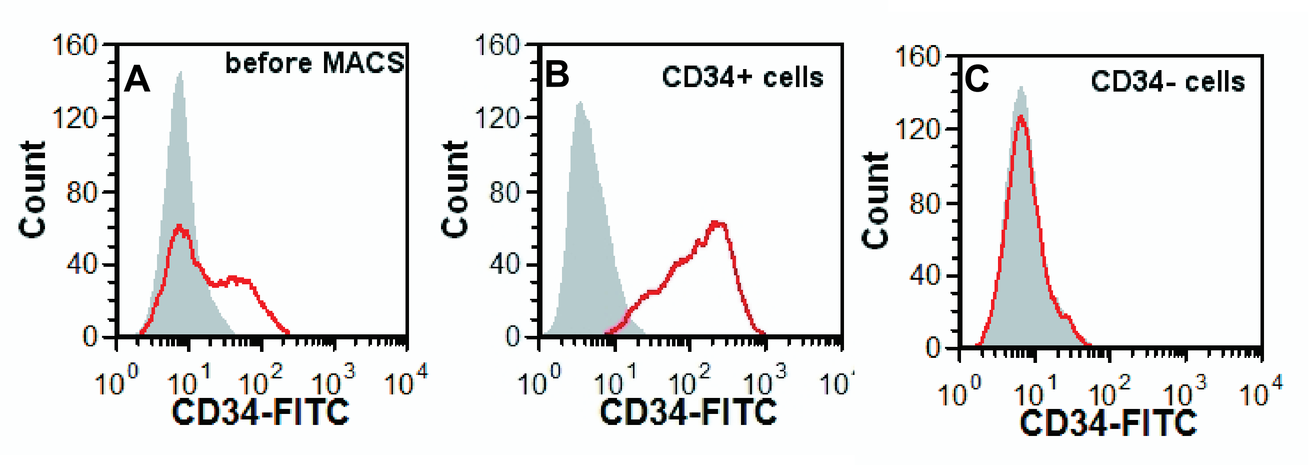

Supp.Fig.2 Expression of CD34 in CD34^{+} and CD34^{-} PB-ECFCs fractions after magnetic separation.

A-C: Representative flow cytometry histograms of the obtained cell population after CD34 labeled magnetic bead separation. Plots depict control isotype IgG staining (grey histograms) versus CD34 antibody staining (empty histograms). **a** depicts a representative histogram of CD34^{+} cells before magnetic separation, **b** represents the number of cells positive for CD34 in the CD34^{+} yielded fraction, **c** depicts the number of cells positive for CD34 in CD34^{-} cells.

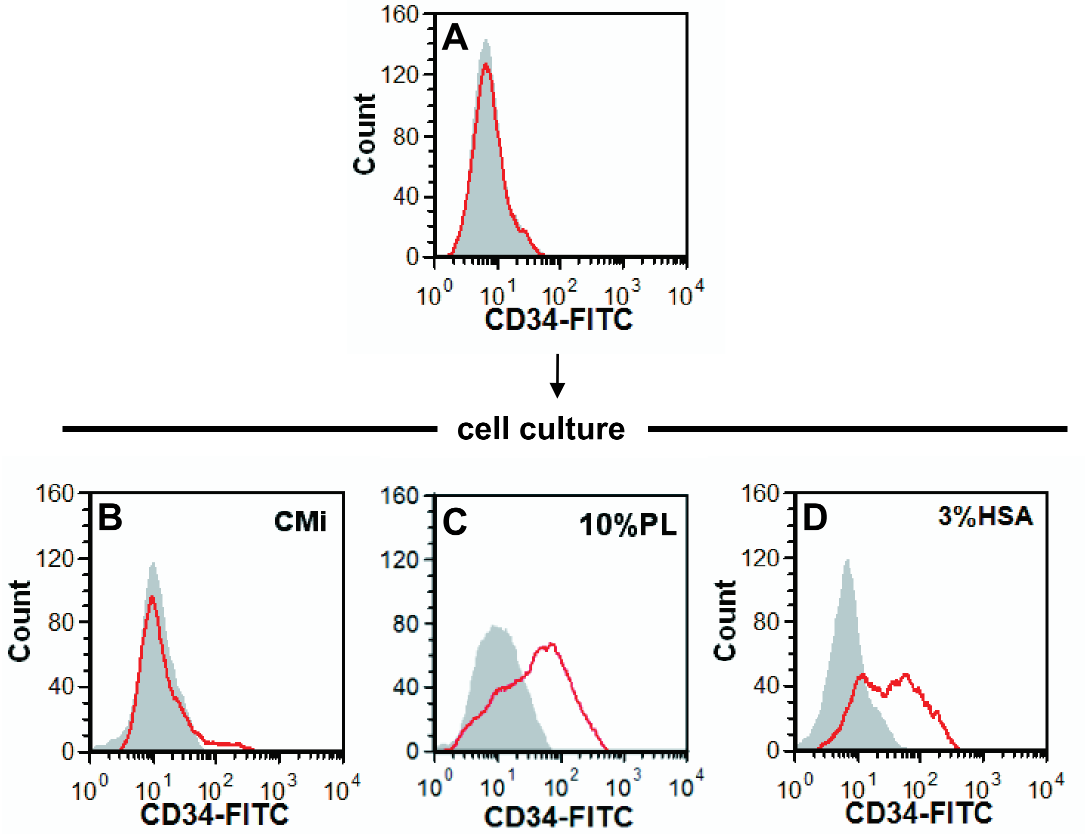

Supp. Fig.3 Flow cytometry histograms of CD34 re- expression in CD34$^-$ ECFCs
a-d: Representative histograms of percentage of cells positive for CD34 when CD34$^-$ cells (**A**) were cultured in CMi (**b**) or PL-EGM (**c**) medium or incubated in medium with 3%HSA for 24h (**d**).

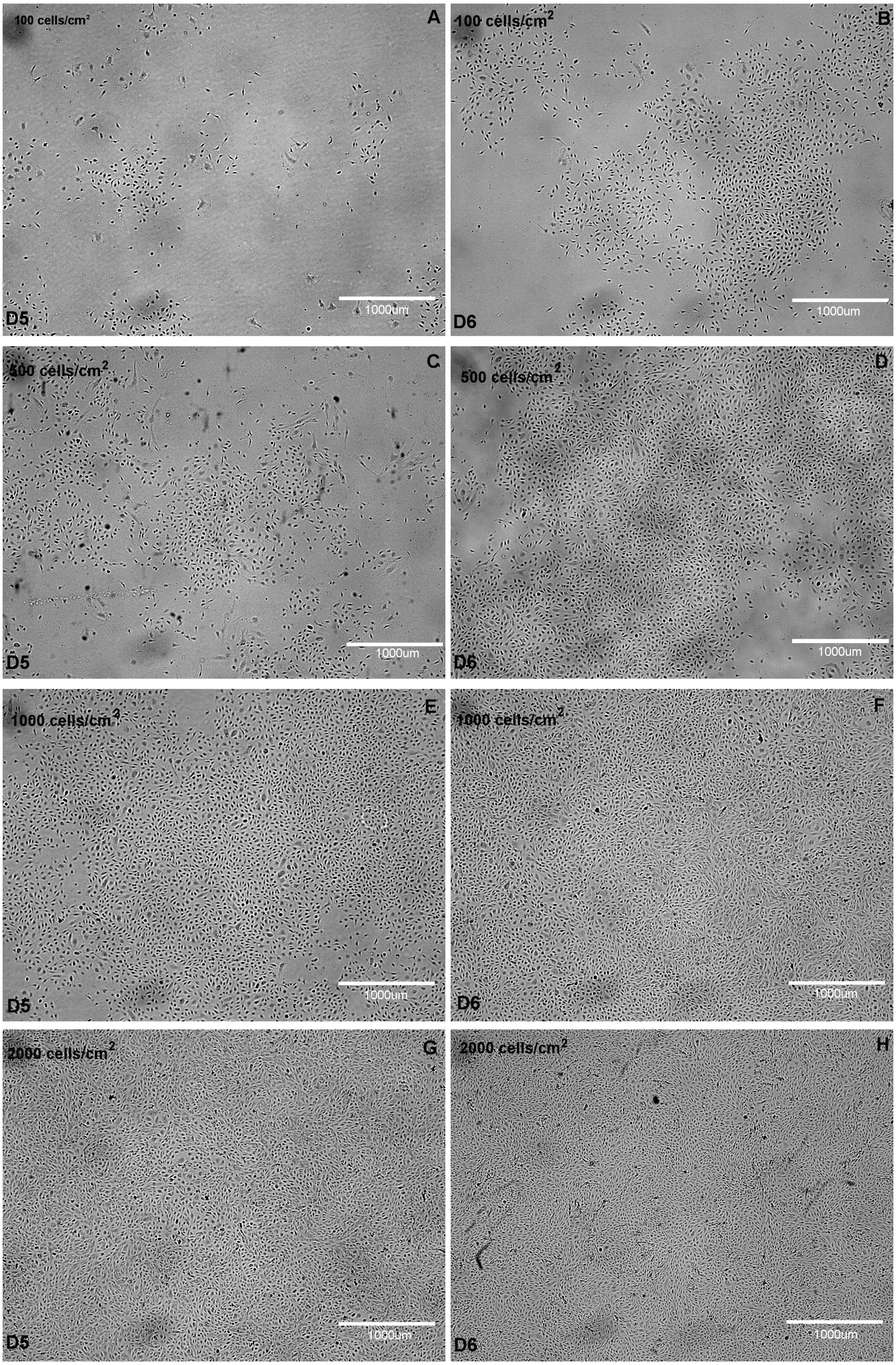

Supp. Fig.4 State of confluence of ECFCs cultures after five and six days proliferation

a-h: Representative phase contrast images of cultures of PB-ECFCs initiated with 100 (**a,b**), 500 (**c,d**), 1000 (**e,f**), and 2000 (**g,h**) cells/cm^2 after five (**D5**) and six (**D6**) days proliferation.

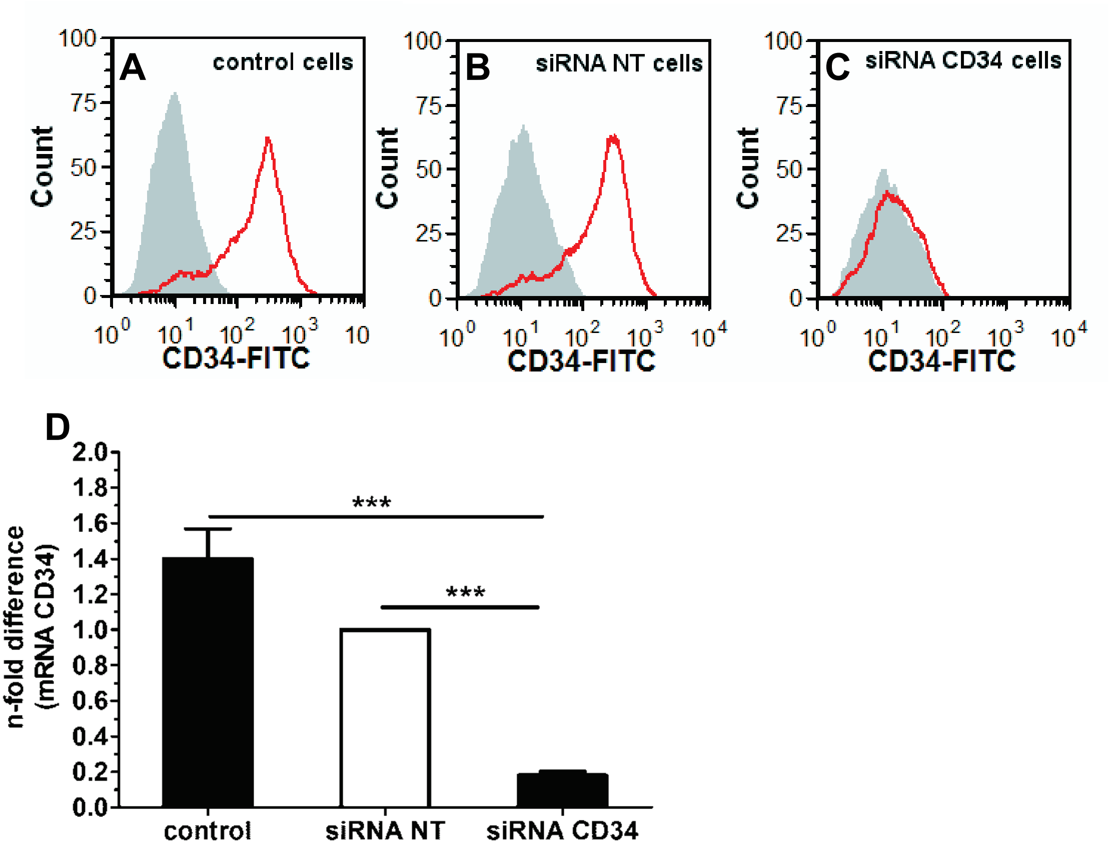

Supp. Fig.5 Evaluation of efficiency of silencing CD34 by siRNA

a: Representative flow cytometry histograms of CD34-FITC positive PB-ECFCs in (A) control, non-transfected cells, (B) in the cells transfected with non-targeting siRNA, and (C) in the cells transfected with siRNA against CD34. Plots depict control isotype IgG staining (grey histograms) versus CD34 antibody staining (empty histograms).

b: qRT-PCR data of efficiency of silencing CD34 with siRNA technology at mRNA level. Data are expressed as a mean ± SEM of n-fold difference of mRNA levels in control and siRNA CD34 transfected cells (closed bars) compared to cell transfected with non-targeting siRNA (open bar).

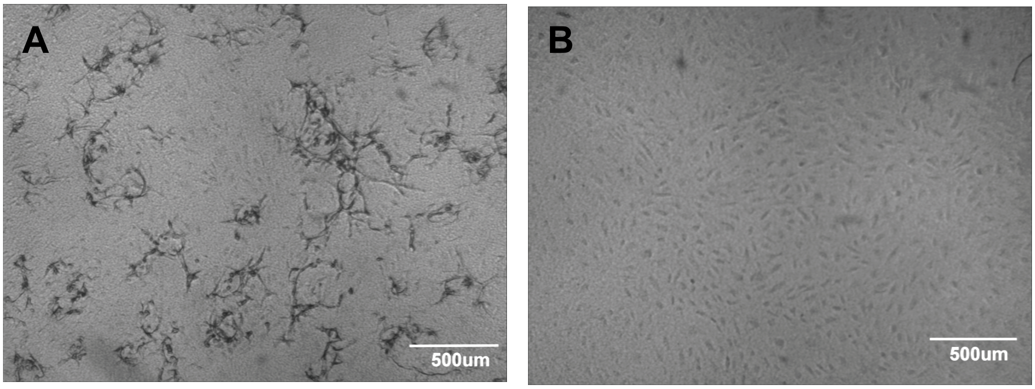

Supp.Fig.6 siRNA uPA abolishes sprouting response of PB-ECFCs in fibrin matrices

a:: Representative phase contrast images of sprout formation in fibrin matrices by control, non-transfected cells upon stimulation with combination of TNF-α and FGF-2.\

b: Representative phase contrast images of absence of sprout formation in fibrin matrices by ECFCs transfected with siRNA u-PA upon stimulation with combination of TNF-α and FGF-2.
